# Prognostic factors in patients with interstitial lung disease treated with nintedanib: a multicenter retrospective study in Japan

**DOI:** 10.1038/s41598-025-34071-7

**Published:** 2025-12-27

**Authors:** Shiho Goda, Tadaaki Yamada, Yasuhiro Goto, Sayaka Uda, Akira Nakao, Shinsuke Shiotsu, Yuji Kukida, Keiko Tanimura, Akifumi Miyamoto, Yuki Imasato, Asuka Okada, Isao Hasegawa, Koji Date, Yohei Matsui, Shoki Morito, Noeru Inoguchi, Shuji Osugi, Hayato Kawachi, Naoya Nishioka, Masahiro Iwasaku, Shinsaku Tokuda, Tomohiro Handa, Koichi Takayama

**Affiliations:** 1 Department of Pulmonary Medicine, Graduate School of Medical science, Kyoto Prefectural University of Medicine, 465 Kajii-cho, Kamigyo-ku, Kyoto, 602-8566 Japan; 2 Department of Respiratory Medicine, Fujita Health University School of Medicine, Toyoake, 470-1192 Japan; 3 Department of Respiratory Medicine, Japanese Red Cross Kyoto Daiichi Hospital, Kyoto, 605-0981 Japan; 4 Department of Respiratory Medicine, Fukuoka University Hospital, Fukuoka, 814-0133 Japan; 5 Department of Respiratory Medicine, Japanese Red Cross Kyoto Daini Hospital, Kyoto, 602-8026 Japan; 6 Department of Rheumatology, Japanese Red Cross Kyoto Daini Hospital, Kyoto, 602-8026 Japan; 7 Department of Respiratory Medicine, Fukuchiyama City Hospital, Fukuchiyama, 620-8505 Japan; 8 Department of Respiratory Medicine, Rakuwakai Otowa Hospital, Kyoto, 607-8062 Japan; 9 Department of Respiratory Medicine, Omi Medical Center, Kusatsu, 525-8585 Japan; 10 Department of Respiratory Medicine, Saiseikai Suita Hospital, Suita, 564-0013 Japan; 11 Department of Respiratory Medicine, Saiseikai Shigaken Hospital, Ritto, 520-3046 Japan; 12 Department of Pulmonary Medicine, Kyoto Chubu Medical Center, Nantan, 629-0197 Japan; 13 Department of Respirology, North Medical Center, Kyoto Prefectural University of Medicine, Yosa, 629-2261 Japan; 14 Department of Respiratory Medicine, Uji Tokushukai Medical Center, Uji, 611-0041 Japan; 15 Department of Respiratory Medicine, Otsu City Hospital, Otsu, 520-0804 Japan; 16 Department of Respiratory Medicine, Japan Community Health Care Organization Kobe Central Hospital, Kobe, 651-1145 Japan; 17 Department of Respiratory Medicine, Graduate School of Medicine, Kyoto University, Kyoto, 606-8507 Japan

**Keywords:** Survival, Prognostic factor, Fibrosis, Interstitial lung disease, Treatment, Nintedanib, Diseases, Medical research

## Abstract

Nintedanib is widely used to slow disease progression and prevent acute exacerbations in patients with idiopathic pulmonary fibrosis (IPF) and progressive fibrosing interstitial lung disease (PF-ILD). We retrospectively analysed patients who initiated nintedanib for IPF or PF-ILD between August 2019 and July 2023 across 15 institutions in Japan, focusing on prognosis and disease progression. Patients were divided into two groups based on whether they survived for ≥ 3 years after nintedanib initiation, and their characteristics were compared. We also evaluated factors associated with annual forced vital capacity (FVC) decline and acute exacerbations. A total of 413 patients (171 with IPF and 242 with PF-ILD) were included. Median survival was 1,177 days for IPF and 1,268 days for PF-ILD, with no significant difference (*P* = 0.20). Patients surviving < 3 years were older (75.0 vs. 71.0 years), more frequently had resting arterial oxygen saturation (SpO_2_) < 95% (44.5% vs. 18.0%), and lower body mass index (BMI) (21.9 vs. 24.1 kg/m^2^). Additionally, patients with a BMI < 22 kg/m^2^ showed greater annual relative FVC decline. In conclusion, patients with IPF and PF-ILD showed comparable outcomes following nintedanib treatment. Age ≥ 65 years, resting SpO_2_ < 95%, and BMI < 22 kg/m^2^ were associated with shorter survival after nintedanib initiation.

**Supplementary Information:**

The online version contains supplementary material available at 10.1038/s41598-025-34071-7.

## Introduction

Interstitial lung disease (ILD) is associated with poor prognosis and high mortality due to progressive pulmonary function decline and acute exacerbations^1,2^.

Standard treatments include glucocorticoids, immunosuppressive agents, and the avoidance of environmental or drug-related causes; however, therapeutic strategies vary by disease subtype and underlying pathophysiology^1,3^.

Idiopathic pulmonary fibrosis (IPF), a subtype of ILD, is characterized by progressive lung fibrosis. The median survival time following diagnosis is only 2‒4 years^4,5^, with acute exacerbations accounting for approximately 40% of deaths^5^. Other ILD subtypes, such as those classified as progressive fibrosing ILD (PF-ILD)^6^ or progressive pulmonary fibrosis (PPF)^1^ also feature progressive fibrosis and functional decline‒particularly in forced vital capacity (FVC)‒despite appropriate treatment. The median survival following disease progression in PF-ILD is 3.7 years, and patients with this condition have a prognosis comparable to that of IPF^7^. Consequently, the development of therapeutic modalities that improve outcomes in IPF and PF-ILD is essential.

Nintedanib is a potent intracellular tyrosine kinase inhibitor that targets receptors for platelet-derived growth factor, fibroblast growth factor, and vascular endothelial growth factor^8^. By inhibiting these receptors, nintedanib suppresses fibroblast proliferation and slows disease progression, establishing its role as a key treatment for ILDs.

In the INPULSIS trials, nintedanib significantly reduced the annual rate of FVC decline in patients with IPF^9,10^. The INBUILD trial subsequently demonstrated similar efficacy in patients with PF-ILD^6^. As a result, nintedanib is now recommended and widely used for the treatment of both IPF and PF-ILD^1,3,11^.

A pooled analysis of the INPULSIS-1 and INPULSIS-2 trials revealed that nintedanib also significantly reduced the risk of first acute exacerbation in patients with IPF^9,12^. Nevertheless, some patients experience disease progression and acute exacerbations during treatment. Reported risk factors for acute exacerbations during nintedanib therapy in IPF include low baseline FVC, home oxygen use, antacid therapy, smoking history at the time of treatment initiation, and shorter treatment duration^9,13^; however, data remain limited. In contrast, while nintedanib has been shown to reduce FVC decline and all-cause mortality in PF-ILD^6,14,15^, data regarding its effect on acute exacerbations in this population are sparse, with only limited evidence suggesting a preventive benefit^16^.

Given these gaps in knowledge, it is clinically important to identify patient characteristics associated with favorable outcomes from nintedanib therapy. Therefore, we aimed to examine the relationship between baseline characteristics and clinical outcomes in patients with IPF and PF-ILD treated with nintedanib, with the goal of identifying prognostic and therapeutic predictors.

## Results

### Patient characteristics

A CONSORT diagram is provided in Supplementary Figure S1. Of the 478 patients initially enrolled, 413 were included in the analysis. We excluded 22 patients who used pirfenidone before or after the initiation of nintedanib, 30 with follow-up duration < 1 year due to reasons other than death, and 13 with systemic sclerosis (SSc) who did not meet the PF-ILD criteria. Patient characteristics are shown in Table 1. The median follow-up period was 739 days. The mean age was 74 (range, 20‒92) years, and 73.1% of patients were male. Among the 413 patients, 171 had IPF and 242 had PF-ILD. Compared with patients with PF-ILD, those with IPF were more likely to be male (88.9% vs. 62.0%), have a history of smoking (78.9% vs. 64.9%), present with a usual interstitial pneumonia pattern on computed tomography (CT) (99.4% vs. 35.5%), and exhibit more severe disease (Stage III–IV: 45.6% vs. 36.4%). However, patients with IPF had lower serum lactate dehydrogenase (LDH) levels (218.5 vs. 229.0 U/L), a lower neutrophil-to-lymphocyte ratio (2.6 vs. 3.0), and were less likely to receive glucocorticoids (19.9% vs. 43.0%) or immunosuppressants (2.9% vs. 26.9%) (shown in Table 1). During the follow-up period, 176 patients died, and 3 underwent lung transplantation.

**Table 1:** Patient characteristics.

	All Patients	Subgroup		*p*-value
		IPF	PF-ILD	
	*n* = 413	*n* = 171	*n* = 242	
Age	74.0 [20‒92]	75.0 [40‒92]	73.0 [20‒91]	0.07
Male	302 (73.1)	152 (88.9)	150 (62.0)	**< 0.001**
BMI, kg/m^2^	22.6 [8.2‒36.8]	22.6 [11.1‒32.4]	22.6 [8.2‒36.8]	0.52
Smoking status				
Former/Current	292 (70.7)	135 (78.9)	157 (64.9)	**< 0.001**
Disease				
IPF	171 (41.4)	171 (100.0)	0 (0)	-
IIPs	109 (26.4)	0 (0)	109 (45.0)	
CTD-ILD	93 (22.5)	0 (0)	93 (38.4)	
PPFE	21 (5.1)	0 (0)	21 (8.7)	
HP	15 (3.6)	0 (0)	15 (6.2)	
CT pattern				
UIP	256 (62.0)	170 (99.4)	86 (35.5)	**< 0.001**
fNSIP	113 (27.4)	1 (0.6)	112 (46.3)	
PPFE	21 (8.7)	0 (0)	21 (8.7)	
cNSIP	3 (1.2)	0 (0)	3 (1.2)	
OP	3 (1.2)	0 (0)	3 (1.2)	
DAD	1 (0.4)	0 (0)	1 (0.4)	
Unclassifiable	16 (6.6)	0 (0)	16 (6.6)	
Past acute exacerbation	64 (15.5)	25 (14.6)	39 (16.1)	0.78
Medication				
Glucocorticoid	138 (33.4)	34 (19.9)	104 (43.0)	**< 0.001**
Immunosuppressant	70 (16.9)	5 (2.9)	65 (26.9)	**< 0.001**
Severity Ⅲ, Ⅳ	166 (40.2)	78 (45.6)	88 (36.4)	**0.007**
mMRC 2‒4	173 (41.9)	69 (40.3)	104 (43.0)	0.36
Resting SpO_2_ < 95% ^a^	123 (29.8)	46 (27.5)	77 (34.5)	0.15
LTOT	74 (17.9)	24 (14.0)	50 (20.7)	0.09
Blood test				
ALB, g/dL	3.9 [2.4‒5.0]	3.9 [2.8‒5.0]	3.9 [2.4‒4.7]	0.51
LDH, U/L	223.0 [139.0‒818.0]	218.5 [141.0‒818.0]	229.0 [139.0‒609.0]	**0.01**
CRP, mg/dL	0.23 [0.00‒25.73]	0.24 [0.01‒25.73]	0.22 [0.00‒12.34]	0.87
KL-6, U/mL	983.0 [160.0‒9450.0]	915.0 [261.0‒4749.0]	1028.0 [160.0‒9450.0]	0.07
NLR	2.8 [0.3‒37.6]	2.6 [0.7‒37.6]	3.0 [0.3‒30.3]	**0.03**
PNI	48.4 [27.5‒71.0]	48.3 [33.2‒60.5]	48.5 [27.5‒71.0]	0.93
Pulmonary function test				
%FVC	70.3 [23.6‒131.2]	71.4 [30.8‒131.2]	69.6 [23.6‒126.6]	0.13
%DLco	56.2 [9.3‒119.8]	53.0 [14.4‒108.0]	57.4 [9.3‒119.80]	0.45
Days from diagnosis to initiation of nintedanib	515.5 [-1164‒6440]	269.5 [-37‒4816]	785.0 [-1164‒6440]	**< 0.001**

Data are expressed as medians [ranges], or N (%). %DLco, % diffusing capacity of the lungs for carbon monoxide; %FVC, % forced vital capacity; ALB, albumin; BMI, body mass index; cNSIP, cellular non-specific interstitial pneumonia; CRP, C-reactive protein; CTD-ILD, connective tissue disease-associated interstitial lung disease; DAD, diffuse alveolar damage; fNSIP, fibrotic non-specific interstitial pneumonia; HP, hypersensitivity pneumonitis; IIPs, idiopathic interstitial pneumonias; IPF, idiopathic pulmonary fibrosis; KL-6, Krebs von den Lungen-6; LDH, lactate dehydrogenase; LTOT, long-term oxygen therapy; mMRC, modified Medical Research Council; NLR, neutrophil-to-lymphocyte ratio; OP, organizing pneumonia; PF-ILD, progressive fibrosing interstitial lung diseases; PNI, prognostic nutritional index; PPFE, pleuroparenchymal fibroelastosis; SpO₂, saturation of arterial oxygen; UIP, usual interstitial pneumonia. ^a^ Those who could not measure their resting SpO_2_ at rest with room air due to oxygen administration were included in resting SpO_2_ < 95%.

### Survival

**Fig. 1:**
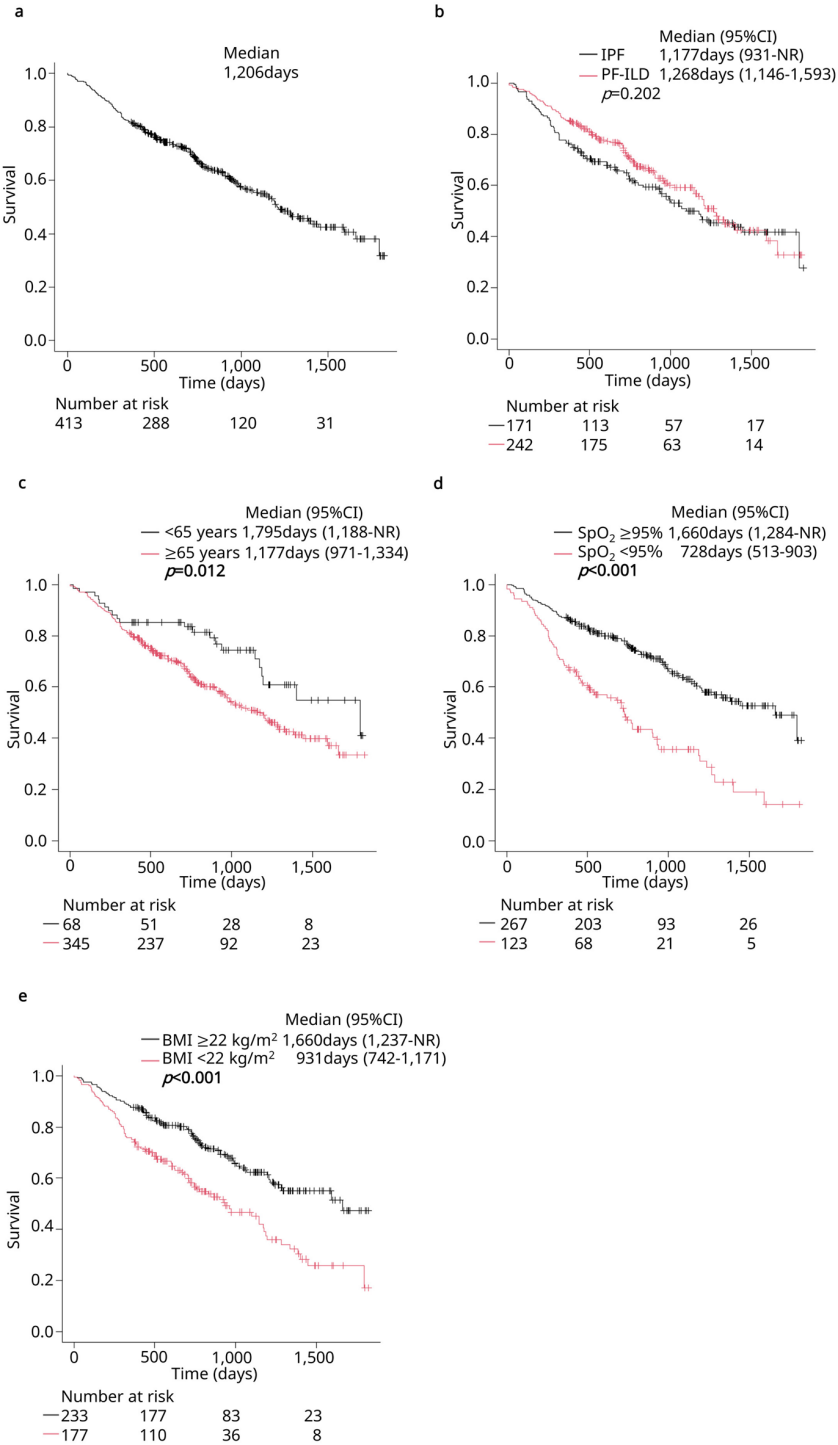
Kaplan–Meier curves of survival after the initiation of nintedanib. **a** Survival in all eligible patients. **b** Survival by diseases. **c** Survival by age. **d** Survival by resting SpO_2_. **e** Survival by BMI. Those whose resting SpO_2_ in room air could not be measured at room air due to oxygen administration were included in the group with resting SpO_2_ < 95%. CI, confidence interval; IPF, idiopathic pulmonary fibrosis; PF-ILD, progressive fibrosing interstitial lung disease; SpO_2_, saturation of arterial oxygen; BMI, body mass index; NR, not reached.

The median survival time following the initiation of nintedanib treatment for all eligible patients was 1,206 days (shown in Fig. 1a, b). Figure 1b shows a comparison of survival between IPF and PF-ILD; the median survival was 1,177 days in IPF and 1,268 days in PF-ILD. There was no significant difference in survival between patients with IPF and those with PF-ILD (*P* = 0.20).

After excluding 152 patients who were lost to follow-up within 3 years for reasons other than death, 261 were divided into two groups based on whether they survived ≥ 3 years after nintedanib initiation. Baseline characteristics were compared between these groups (shown in Table 2). Compared to those who survived ≥ 3 years after nintedanib initiation, patients who survived < 3 years had the following characteristics: older age (75.0 vs. 71.0 years), lower body mass index (BMI) (21.9 vs. 24.1 kg/m^2^), higher smoking history (77.4% vs. 67.0%), more frequent glucocorticoid use (36.8% vs. 24.5%), more severe disease (Stage III–IV: 49.7% vs. 36.8%), higher modified medical research council (mMRC) scores (2–4: 50.8% vs. 36.8%), lower resting arterial oxygen saturation (SpO_2_) (SpO_2_ < 95% or oxygen use at rest: 44.5% vs. 18.0%), more frequent long-term oxygen therapy (LTOT) (25.8% vs. 10.4%), lower serum albumin levels (3.7 vs. 4.0 g/dL), higher serum LDH levels (234.0 vs. 218.0U/L), higher serum C-reactive protein levels (0.37 vs. 0.19 mg/dL), and lower %FVC (60.9% vs. 75.7%). Conversely, sex, disease, and CT patterns did not differ significantly between the two groups. The results of multivariate analysis for items that differed significantly between the two groups are shown in Table 3. Older age, low resting SpO_2_, and low BMI were the independent factors predicting survival of < 3 years after the initiation of nintedanib. A subgroup analysis of survival was performed in 413 patients using three prognostic factors: age, resting SpO_2_, and BMI (shown in Fig. 1c-e). Survival was significantly shorter for those aged ≥ 65 years, resting SpO_2_ < 95%, and BMI < 22 kg/m^2^.

**Table 2:** Patient characteristics of survival time.

	Survival < 3 year	Survival ≥ 3 year	*p*-value
	*n* = 155	*n* = 106	
Age	75.0 [20‒92]	71.0 [40‒80]	**< 0.001**
Male	125 (80.6)	68 (64.2)	0.16
BMI, kg/m^2^	21.9 [8.2‒31.5]	24.1 [11.1‒32.6]	**< 0.001**
Smoking status			
Former/Current	120 (77.4)	71 (67.0)	**0.04**
Disease			
IPF	75 (48.4)	51 (48.1)	0.96
IIPs	36 (23.2)	24 (22.6)	
CTD-ILD	28 (18.1)	21 (19.8)	
PPFE	9 (5.8)	7 (6.6)	
HP	5 (3.2)	3 (2.8)	
CT pattern			
UIP	106 (68.4)	68 (64.2)	0.88
fNSIP	34 (21.9)	26 (24.5)	
PPFE	8 (5.2)	7 (6.6)	
Unclassifiable	7 (4.5)	5 (4.7)	
Past acute exacerbation	29 (18.7)	14 (13.2)	0.31
Medication			
Glucocorticoid	57 (36.8)	26 (24.5)	**0.04**
Immunosuppressant	24 (15.5)	15 (14.2)	0.86
Severity Ⅲ, Ⅳ	77 (49.7)	36 (36.8)	**0.01**
mMRC 2‒4	78 (50.8)	36 (36.8)	**0.001**
Resting SpO_2_ < 95% ^a^	69 (44.5)	19 (18.0)	**< 0.001**
LTOT	40 (25.8)	11 (10.4)	**0.002**
Blood test			
ALB, g/dL	3.7 [2.4‒5.0]	4.0 [3.1‒4.6]	**< 0.001**
LDH, U/L	234.0 [139.0‒818.0]	218.0 [142.0‒401.0]	**0.004**
CRP, mg/dL	0.37 [0.01‒8.02]	0.19 [0.00‒25.73]	**< 0.001**
KL-6, U/mL	926.0 [261.0‒5570.0]	997.0 [160.0‒4749.0]	0.97
NLR	2.6 [0.72‒37.60]	2.9 [0.3‒25.4]	0.08
PNI	48.7 [33.2‒60.5]	48.0 [27.5‒71.0]	0.21
Pulmonary function test			
%FVC	60.9 [23.6‒112.7]	75.7 [37.3‒131.2]	**< 0.001**
%DLco	52.7 [14.4‒112.6]	58.6 [30.2‒108.0]	0.15
Days from diagnosis to initiation of nintedanib	588.0 [-302‒5596]	220.0 [-1166‒5660]	0.07

Data are expressed as medians [ranges], or N (%). %DLco, % diffusing capacity of the lungs for carbon monoxide; %FVC, % forced vital capacity; ALB, albumin; BMI, body mass index; CRP, C-reactive protein; CTD-ILD, connective tissue disease-associated interstitial lung disease; fNSIP, fibrotic non-specific interstitial pneumonia; HP, hypersensitivity pneumonitis; IIPs, idiopathic interstitial pneumonias; IPF, idiopathic pulmonary fibrosis; KL-6, Krebs von den Lungen-6; LDH, lactate dehydrogenase; LTOT, long-term oxygen therapy; mMRC, modified Medical Research Council; NLR, neutrophil-to-lymphocyte ratio; PNI, prognostic nutritional index; PPFE, pleuroparenchymal fibroelastosis; SpO₂, saturation of arterial oxygen; UIP, usual interstitial pneumonia. ^a^ Those who could not measure their resting SpO_2_ with room air due to oxygen administration were included in resting SpO_2_ < 95%.

**Table 3:** Multivariate analysis of patient characteristics predicting survival.

	Odds ratio	95% confidence interval	*p*-value
Age	0.919	0.87‒0.97	**0.03**
BMI	1.210	1.05‒1.40	**0.01**
Smoking experience	0.321	0.10‒1.03	0.057
Severity Ⅲ, Ⅳ	0.440	0.12‒1.66	0.23
mMRC 2‒4	1.980	0.56‒6.98	0.29
Resting SpO_2_ < 95% ^a^	0.225	0.06‒0.85	**0.03**
LTOT	1.610	0.38‒6.88	0.52
Serum ALB	0.745	0.18‒3.12	0.69
Serum LDH	0.993	0.98‒1.00	0.17
Serum CRP	1.170	0.61‒2.25	0.64
%FVC	1.040	0.99‒1.08	0.06

%FVC, % forced vital capacity; ALB, albumin; BMI, body mass index; CRP, C-reactive protein; LDH, lactate dehydrogenase; LTOT, long-term oxygen therapy; mMRC, modified Medical Research Council; SpO₂, saturation of arterial oxygen. ^a^ Those who could not measure their resting SpO_2_ with room air due to oxygen administration were included in resting SpO_2_ < 95%.

Furthermore, a subgroup analysis was performed by dividing the patients into two groups: patients with IPF and patients with PF-ILD. Baseline characteristics of patients with IPF and PF-ILD patients were compared between the survival time groups (shown in Supplementary Table S1 and Supplementary Table S2). Both patients with IPF and PF-ILD generally showed similar characteristics to the overall population. Additionally, the impact of the three prognostic factors identified in the overall population analysis (age, resting SpO₂, BMI) on survival duration were examined for both patients with IPF and patients with PF-ILD (shown in Supplementary Fig. S2). Although the number of younger patients with IPF was very small and no difference was observed compared to older patients with IPF, other factors showed similar trends.

### FVC decline

**Fig. 2:**
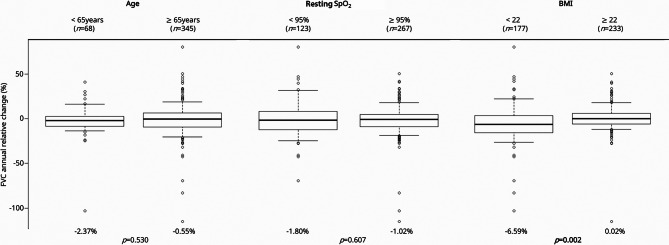
Forced vital capacity annual relative decline in subgroup analysis. SpO_2_, saturation of arterial oxygen; BMI, body mass index. Those whose resting SpO_2_ in room air cannot be measured at room air due to oxygen administration are included in the group with resting SpO_2_ < 95%.

Patients whose survival was < 3 years after the initiation of nintedanib had a significantly greater FVC decline (shown in Table 4). Thus, the factors associated with annual FVC change after the initiation of nintedanib were examined. First, the annual relative change in FVC was compared among the 413 patients included in the analysis, using three prognostic factors: age, resting SpO_2_, and BMI (shown in Fig. 2). While age and resting SpO_2_ did not affect the rate of FVC decline, patients with BMI < 22 kg/m^2^ had a significantly greater relative annual FVC decline (-6.59% vs. 0.02%). A subgroup analysis was performed by dividing the patients into two groups: patients with IPF and patients with PF-ILD. In the patients with IPF, FVC decline was significantly greater in patients with lower BMI, while no significant differences were observed in other factors. Overall, the results were generally consistent with those of the overall population (shown in Supplementary Fig. S3). Second, other factors affecting the relative annual changes in FVC were analyzed. Of the 413 patients included in the analysis, 214 with data on annual relative FVC change data had a median change of -1.37%. A univariate analysis of patient characteristics was performed for two groups stratified by FVC annual relative change (≥-1.37% or <-1.37%). The group with a greater FVC annual relative decline was significantly more likely to have a lower BMI (23.9 vs. 22.3 kg/m^2^) and to be non-users of immunosuppressants (21.5% vs. 9.3%) (shown in Table S3).

**Table 4:** Progress of interstitial lung disease after initiation of nintedanib.

	Survival < 3 year	Survival ≥ 3 year	*p*-value
	*n* = 155	*n* = 106	
FVC annual change, mL/year	-139.5 [-2022‒1303]	-45.74 [-780‒575]	**0.006**
FVC annual relative change, %	-7.3 [-115.2‒80.3]	-1.4 [-23.0‒50.1]	**0.005**
Acute exacerbation within 3 years	56 (36.1)	19 (17.9)	**0.001**

Data are expressed as medians [ranges], or N (%). FVC, forced vital capacity.

### Acute exacerbations

Of the 426 patients, 99 experienced acute exacerbations during the observation period following the initiation of nintedanib. The median time from nintedanib initiation to the first acute exacerbation was 444 days, and the median survival after the first acute exacerbation was 95 days (*n* = 413). There was no significant difference in the time to acute exacerbation between patients with IPF (431 days) and those with PF-ILD (451 days). Among the 261 patients with ≥ 3 years of follow-up, acute exacerbations were significantly more frequent in those who survived < 3 years (36.1% vs. 17.9%, Table 4). The cumulative incidence of acute exacerbations was analysed based on three prognostic factors—age, resting SpO_2_, and BMI—none of which showed significant differences (shown in Table 5). The results of subgroup analyses in patients with IPF and PF-ILD were consistent with those in the overall population, and the three prognostic factors did not affect the cumulative incidence rate of acute exacerbations (data not shown). In a subset of 176 patients who could be followed until death, those who experienced acute exacerbation within 3 years of nintedanib initiation were significantly more likely to have a history of acute exacerbations and a higher BMI (shown in Table S4). Multivariate analysis of factors that significantly differed between the groups (shown in Table S5) identified both a history of acute exacerbations and high BMI as independent predictors of future exacerbation following nintedanib initiation.

**Table 5:** Analysis of potential risk factors on the cumulative incidence of acute exacerbations.

	Hazard ratio	95% confidence interval	*p*-value
Age ≥ 65 years	1.479	0.821‒2.666	0.19
Resting SpO_2_ < 95% ^a^	1.240	0.823‒1.881	0.30
BMI ≥ 22 kg/m^2^	1.093	0.737‒1.620	0.66

The analysis was performed using the Fine‒Gray test.

^a^ Those who could not measure their resting SpO_2_ with room air due to oxygen administration were included in resting SpO_2_ < 95%.

### Nintedanib usage conditions

Nintedanib use was compared between groups based on whether patients survived ≥ 3 years after treatment initiation (shown in Table S6). Patients who survived < 3 years were more likely to have received the maximum dose of 200 mg and had a higher discontinuation rate (44.5% vs. 29.2%). The duration of nintedanib therapy was significantly shorter in patients who survived < 3 years (257 vs. 1,326 days). However, the reasons for discontinuing nintedanib did not differ significantly between the groups. Furthermore, when examining each prognostic factor separately, the maximum dose of 200 mg/day was significantly more common in patients aged ≥ 65 years (34.8% vs. 22.1%), with resting SpO₂ <95% (34.1% vs. 32.2%), and BMI < 22 kg/m2 (39.0% vs. 27.9%). The duration of nintedanib therapy was also significantly shorter in patients aged ≥ 65 years (539 vs. 841 days), with resting SpO₂ <95% (426 vs. 657 days), and BMI < 22 kg/m2 (462 vs. 703 days). A multivariate analysis was performed to assess the effects of nintedanib dose and treatment duration on survival, FVC decline, and the occurrence of acute exacerbations. None of these factors were identified as independent prognostic factors (data not shown).

## Discussion

This study retrospectively followed the long-term clinical course of over 400 patients with ILD receiving nintedanib. To the best of our knowledge, this is the first report to directly compare the clinical outcomes of patients with IPF and PF-ILD following nintedanib initiation in real-world clinical practice and to demonstrate comparable outcomes between the two groups. Survival and FVC decline after treatment initiation were comparable to previously reported findings^3,17–21^. The incidence of acute exacerbations was higher than in prior clinical trials, likely owing to the inclusion of patients with poorer pulmonary function who would have been ineligible for those trials^16^. In the overall population, older age, resting SpO_2_ <95%, and lower BMI were associated with shorter survival after the initiation of nintedanib. Similar results were generally observed in the subgroup analyses of patients with IPF and PF-ILD. Lower BMI and use of immunosuppressants were also associated with a greater FVC decline after nintedanib initiation. A history of acute exacerbation was a predictive factor for acute exacerbation within 3 years. Although high BMI was also suspected to be the predictive factors of acute exacerbation within 3 years after the initiation of nintedanib in multivariate analysis (shown in Supplementary table S5), BMI was not a significant factor in the cumulative incidence analysis (shown in Table 5), which included a larger sample size. This discrepancy warrants further investigation.

There was no significant difference in survival after nintedanib initiation between patients with IPF and those with PF-ILD, in line with earlier studies^22^. Disease subtype or CT pattern was not associated with survival. Therefore, patients with PF-ILD should be treated with nintedanib as proactively as those with IPF, given the similarity in clinical course.

Due to limited prognostic data for PF-ILD, we aimed to identify relevant predictive factors. Older patients demonstrated significantly shorter survival; however, FVC decline was comparable between age groups. Previous studies have shown that nintedanib reduces annual FVC decline in both older and younger patients with IPF and PF-ILD^23–25^. Moreover, it may prolong survival and reduce the incidence of acute exacerbations in older patients with IPF^13^. While older individuals more frequently report adverse events, such as diarrhea, nausea, and liver dysfunction^24^, tolerability and treatment continuation rates appear comparable to those in younger patients^24,26^. Thus, nintedanib should be used in older patients with appropriate management to prevent adverse effects.

Patients with ILD who receive LTOT for chronic hypoxemia have significantly shorter survival times than those without hypoxemia^27–29^. Similarly, patients with PF-ILD hypoxemia show significantly poorer survival outcomes^30^. In contrast, antifibrotic agents have been reported to significantly improve life expectancy in patients with IPF on LTOT^31^. In this study, patients with hypoxemia had a poor prognosis after antifibrotic therapy initiation, likely reflecting underlying disease severity rather than treatment failure. Early initiation of antifibrotic agents‒prior to the onset of hypoxemia‒may improve outcomes in PF-ILD, as seen in IPF^32,33^.

Lower BMI was significantly associated with both shorter survival and greater FVC decline in patients with IPF and associated to shorter survival in patients with PF-ILD. These findings align with previous reports indicating that patients with IPF and PF-ILD with low BMI experience greater FVC decline^34,35^ and higher mortality^35–39^. Antifibrotic agents have demonstrated efficacy regardless of BMI in both IPF and PF-ILD^34,35^, and we believe that further prognostic improvement can be expected with the use of nintedanib to manage low body weight. As nintedanib often causes diarrhea and anorexia, managing adverse effects and prevention of weight loss are essential^40^. Although there is no established nutritional therapy for ILD, guidelines for chronic obstructive pulmonary disease (COPD) recommend a balanced macronutrient intake (15–20% proteins, 30–45% fat, 40–55% carbohydrates), 4–6 small frequent meals, dietary supplements, and intake of omega-3 polyunsaturated fatty acids and vitamin D^41^. Some reports have shown that nutritional therapy based on COPD recommendations are beneficial for patients with ILD^42^, and similar strategies may support patients with ILD with low BMI.

In this study, patients who survived < 3 years were more likely to discontinue nintedanib early, resulting in significantly shorter treatment durations. However, reasons for discontinuation did not differ significantly between groups, and adverse effects were not notably more frequent. Patients aged ≥ 65 years, with resting SpO₂ <95%, or BMI < 22 kg/m² often receive lower doses of nintedanib or shorter treatment durations, which may be associated with short survival. Long-term use of nintedanib reportedly improves survival and reduces the risk of acute exacerbation^13^. Therefore, sustained treatment is important to optimizing outcomes.

Patients with IPF and PF-ILD were enrolled based on criteria consistent with insurance coverage for nintedanib in Japan. While PF-ILD and PPF are closely related, future studies should focus specifically on PPF. Additionally, a third antifibrotic agent has recently demonstrated efficacy in both IPF and PPF^43,45^. Continued development and clinical evaluation of new antifibrotic treatments—including combination and sequential therapy strategies—are warranted.

This study has several limitations. First, it was a non-randomized, retrospective analysis and may be subject to bias in data collection and treatment selection. Second, many patients had missing data—particularly for diffusing capacity of the lungs for carbon monoxide (DLco), partial pressure of arterial oxygen (PaO_2_), mMRC, and 6-minute walk test (6MWT)—which limited the assessment of ILD severity. Since we did not investigate the reasons for the missing data, it is possible that cases in which the condition was severe and testing was not possible were omitted from the analysis. Third, variability in CT interpretation between institutions may have affected diagnostic accuracy. Fourth, although glucocorticoids may affect prognosis and acute exacerbation incidence in patients with IPF, confounding factors and small sample size prevented a definitive assessment.

In conclusion, survival following nintedanib initiation was comparable between patients with IPF and those with PF-ILD. Older age, lower resting SpO_2_, and lower BMI were associated with shorter survival. Lower BMI was linked to greater FVC decline. Early initiation of nintedanib and optimization of nutritional status may improve prognosis in both IPF and PF-ILD. Future studies should prospectively examine the impact of early nintedanib initiation and explore the role of nutritional interventions and adverse effect management in improving survival and lung function, particularly among patients with low BMI or advanced age.

## Methods

### Patients

We retrospectively enrolled patients who began nintedanib treatment between August 2019 and July 2023 for either IPF or PF-ILD at 15 institutions in Japan (University Hospital Kyoto Prefectural University of Medicine, Fujita Health University Hospital, Japanese Red Cross Kyoto Daiichi Hospital, Fukuoka University Hospital, Japanese Red Cross Kyoto Daini Hospital, Fukuchiyama City Hospital, Rakuwakai Otowa Hospital, Omi Medical Center, Saiseikai Suita Hospital, Saiseikai Shigaken Hospital, Kyoto Chubu Medical Center, North Medical Center Kyoto Prefectural University of Medicine, Uji Tokushukai Medical Center, Otsu City Hospital, and Japan Community Health Care Organization Kobe Central Hospital). Eligible patients were followed up for ≥ 1 year after initiating nintedanib. In this retrospective study, we reviewed the medical records of the patients and collected the following data from the first visit to July 2024: age, sex, height, weight, smoking status, type of ILD, CT pattern, days from ILD diagnosis to the initiation of nintedanib, LTOT at initiation of nintedanib, respiratory symptoms, SpO_2_, PaO_2_, mMRC dyspnea scale, 6MWT, FVC and DLco (2 years before, at initiation, and 1 year after initiation of nintedanib), vital capacity (at initiation of nintedanib), blood laboratory findings, baseline glucocorticoid or immunosuppressive drug use and dose, date of start and discontinuation of nintedanib, starting and maximum dose, type and severity of nintedanib side effects, time to first acute exacerbation, respiratory-related emergency hospitalization, and death from the initiation of nintedanib. The type of ILD was diagnosed based on the judgment of at least two respiratory physicians: the attending physician and the data collection physician in each case. CT patterns were assessed by at least two respiratory physicians or by one respiratory physician and one radiologist at each institution; in cases of disagreement, consensus was reached through consultation. In patients receiving nintedanib for PF-ILD, central adjudication confirmed that at least one of the following INBUILD trial criteria was met within 24 months prior to starting nintedanib: a relative decline in FVC ≥ 10% predicted; a relative decline in FVC of 5‒<10% predicted accompanied by worsened respiratory symptoms; a relative decline in FVC of 5‒<10% predicted and increased extent of fibrosis on CT; or worsened respiratory symptoms and increased extent of fibrosis on CT^6,45^. This central adjudication was based on reports from each institution regarding the presence or absence of progression in symptom and fibrosis on CT progression, as well as results of pulmonary function test. SSc patients were evaluated by central adjudication to determine whether they met the criteria for PF-ILD, and those who did not meet the criteria were excluded from the analysis.

Annual relative FVC decline and absolute FVC reduction after the initiation of nintedanib were calculated based on pulmonary function test results at the start of treatment and at the time point closest to 1 year after initiation. Disease severity was assessed using the Japanese severity staging system for idiopathic interstitial pneumonias (revised April 2024), defined as follows: stage I, PaO_2_ at rest ≥ 80 Torr and minimum SpO_2_ during 6MWT ≥ 90%; stage II, PaO_2_ at rest 70‒<80 Torr and minimum SpO_2_ during 6MWT ≥ 90%; stage III, PaO_2_ at rest 60‒<70 Torr and minimum SpO_2_ during 6MWT ≥ 90%, or PaO_2_ at rest ≥ 70 Torr and minimum SpO_2_ during 6MWT < 90%; stage IV, PaO_2_ at rest < 60 Torr, or PaO_2_ at rest 60‒<70 Torr and minimum SpO_2_ during 6MWT < 90%.

Acute exacerbations were defined as worsening respiratory symptoms and oxygenation within one month, worsening bilateral chest imaging findings, initiation or increase in glucocorticoid use, and exclusion of heart failure.

Since the median survival time for IPF and PF-ILD is reported to be around three years^4,5,7^, and the median survival time in this study was about three years, we decided to investigate the factors that affect survival time using three years as the cutoff point. Comparisons were made between the two groups for age, resting SpO_2_, and BMI using 65 years, 95%, and 22 kg/m^2^ as cutoff values. The BMI cutoff was calculated and determined based on the ROC curve (shown in Fig. S4).

This study adhered to the Declaration of Helsinki. The study protocol was approved by the Ethics Committee of the Kyoto Prefectural University of Medicine (No. ERB-C-3278). The need for informed consent was waived due to the retrospective nature of the study, and an opt-out opinion was made available on the institution website, as approved by the ethics committee of the Kyoto Prefectural University of Medicine. Approval was also obtained from the ethics committees of all participating institutions.

### Statistical analysis

Statistical analyses were conducted using EZR software (version 4.3.1; Saitama Medical Center, Jichi Medical University; Saitama, Japan)^46^. Survival curves were generated using the Kaplan–Meier method, and differences were analyzed with the log-rank test. The cumulative incidence of acute exacerbations was analyzed using the Fine-Gray test, and differences were assessed using the log-rank test. Fisher’s exact test and Mann-Whitney U test for univariate analysis and multiple regression analysis for multivariate analysis were used to compare background factors. The significance threshold was set at *P* < 0.05. Missing data were excluded from the analysis only for the relevant variables. Reference values for each test followed the standards adopted by each institution.

## Supplementary Information

Below is the link to the electronic supplementary material.

Supplementary Material 1

## Data Availability

All data were generated or analyzed during the current study. Requests for further information can be directed to the corresponding author.
