# Supplementary material for: Prognostic factors in patients with interstitial lung disease treated with nintedanib: a multicenter retrospective study in Japan
Source: Sci Rep. 2025 Dec 27;16:3947. doi: 10.1038/s41598-025-34071-7 (PMC12855916; doi:10.1038/s41598-025-34071-7)

**Table S1.** Patient characteristics of survival time for patients with IPF

|  | **Survival <3 year** | **Survival ≥3 year** | **p-value** |
| --- | --- | --- | --- |
|  | **n=75** | **n=51** |  |
| **Age** | 76.0 [48‒92] | 73.0 [40‒83] | **0.01** |
| **Male** | 68 (90.7) | 45 (88.2) | 0.77 |
| **BMI, kg/m^2^** | 21.4 [13.7‒31.5] | 24.8 [11.1‒32.4] | **<0.001** |
| **Smoking status** |  |  |  |
| **Former/Current** | 63 (84.0) | 38 (74.5) | 0.27 |
| **Past acute exacerbation** | 11 (14.7) | 7 (13.7) | 1.00 |
| **Medication** |  |  |  |
| **Glucocorticoid** | 19 (25.3) | 3 (5.9) | **0.004** |
| **Immunosuppressant** | 3 (4.0) | 0 (0.0) | 0.27 |
| **Severity Ⅲ, Ⅳ** | 39 (52.0) | 20 (39.2) | **0.047** |
| **mMRC 2‒4** | 33 (44.0) | 16 (31.3) | 0.06 |
| **Resting SpO_2_ <95% ^a^** | 30 (40.0) | 7 (13.7) | **0.003** |
| **LTOT** | 17 (23.0) | 1 (2.0) | **0.001** |
| **Blood test** |  |  |  |
| **ALB, g/dL** | 3.8 [2.9‒5.0] | 4.1 [3.2‒4.6] | **<0.001** |
| **LDH, U/L** | 225.0 [141.0‒818.0] | 212.5 [143.0‒346.0] | **0.037** |
| **CRP, mg/dL** | 0.43 [0.01‒8.02] | 0.22 [0.03‒25.73] | **0.012** |
| **KL-6, U/mL** | 850.0 [261.0‒3814.0] | 1009.0 [349.0‒4749.0] | 0.41 |
| **NLR** | 3.0 [0.72‒37.60] | 2.2 [1.0‒8.4] | **0.01** |
| **PNI** | 47.0 [33.2‒60.5] | 50.9 [36.8‒59.9] | **0.001** |
| **Pulmonary function test** |  |  |  |
| **%FVC** | 63.4 [30.8‒109.8] | 81.1 [37.3‒131.2] | **0.001** |
| **%DLco** | 50.2 [14.4‒80.1] | 61.4 [30.2‒108.0] | **0.047** |
| **Days from diagnosis to initiation of nintedanib** | 477.0 [0‒3549] | 66.0 [0‒3036] | **<0.001** |

Data are expressed as medians [ranges], or N (%).

%DLco, % diffusing capacity of the lungs for carbon monoxide; %FVC, % forced vital capacity; ALB, albumin; BMI, body mass index; CRP, C-reactive protein; IPF, idiopathic pulmonary fibrosis; KL-6, Krebs von den Lungen-6; LDH, lactate dehydrogenase; LTOT, long-term oxygen therapy; mMRC, modified Medical Research Council; NLR, neutrophil-to-lymphocyte ratio; PNI, prognostic nutritional index; SpO₂, saturation of arterial oxygen.

^a^ Those who could not measure their resting SpO_2_ with room air were included in resting SpO_2_ <95%

**Table S2.** Patient characteristics of survival time for patients with PF-ILD

|  | **Survival <3 year** | **Survival ≥3 year** | **p-value** |
| --- | --- | --- | --- |
|  | **n=80** | **n=55** |  |
| **Age** | 74.0 [20‒91] | 69.0 [40‒84] | **0.001** |
| **Male** | 57 (71.2) | 32 (58.2) | 0.14 |
| **BMI, kg/m^2^** | 22.1 [8.2‒29.9] | 23.3 [13.3‒32.6] | **0.03** |
| **Smoking status** |  |  |  |
| **Former/Current** | 57 (71.3) | 33 (60.0) | 0.13 |
| **Disease** |  |  |  |
| **IIPs** | 36 (45.0) | 24 (43.6) | 0.91 |
| **CTD-ILD** | 28 (35.0) | 21 (38.2) |  |
| **PPFE** | 9 (11.2) | 7 (12.7) |  |
| **HP** | 5 (6.2) | 3 (5.5) |  |
| **CT pattern** |  |  |  |
| **UIP** | 32 (40.0) | 17 (30.9) | 0.76 |
| **fNSIP** | 33 (41.2) | 26 (47.3) |  |
| **PPFE** | 8 (10.0) | 7 (12.7) |  |
| **Unclassifiable** | 7 (8.8) | 5 (9.1) |  |
| **Past acute exacerbation** | 18 (22.5) | 7 (12.7) | 0.18 |
| **Medication** |  |  |  |
| **Glucocorticoid** | 38 (47.5) | 23 (41.8) | 0.60 |
| **Immunosuppressant** | 21 (26.2) | 15 (27.3) | 1.00 |
| **Severity Ⅲ, Ⅳ** | 77 (96.3) | 36 (65.5) | **0.01** |
| **mMRC 2‒4** | 38 (47.5) | 16 (29.1) | 0.28 |
| **Resting SpO_2_ <95% ^a^** | 39 (48.8) | 12 (21.8) | **0.001** |
| **LTOT** | 23 (28.7) | 10 (18.2) | 0.22 |
| **Blood test** |  |  |  |
| **ALB, g/dL** | 3.7 [2.4‒4.6] | 4.0 [3.1‒4.6] | **0.001** |
| **LDH, U/L** | 249.0 [139.0‒609.0] | 220.0 [149.0‒401.0] | **0.04** |
| **CRP, mg/dL** | 0.32 [0.01‒4.25] | 0.18 [0.00‒12.34] | **0.01** |
| **KL-6, U/mL** | 962.0 [310.0‒5570.0] | 983.0 [160.0‒2579.0] | 0.50 |
| **NLR** | 3.2 [0.33‒25.4] | 2.7 [0.8‒14.4] | 0.27 |
| **PNI** | 44.9 [27.5‒71.0] | 49.9 [37.1‒61.2] | **0.001** |
| **Pulmonary function test** |  |  |  |
| **%FVC** | 59.3 [23.6‒112.7] | 73.5 [46.1‒116.9] | **0.005** |
| **%DLco** | 53.2 [23.7‒112.6] | 54.4 [33.1‒96.6] | 0.91 |
| **Days from diagnosis to initiation of nintedanib** | 742.0 [-302‒5096] | 900.0 [-1164‒5660] | 0.34 |

Data are expressed as medians [ranges], or N (%).

%DLco, % diffusing capacity of the lungs for carbon monoxide; %FVC, % forced vital capacity; ALB, albumin; BMI, body mass index; CRP, C-reactive protein; CTD-ILD, connective tissue disease-associated interstitial lung disease; fNSIP, fibrotic non-specific interstitial pneumonia; HP, hypersensitivity pneumonitis; IIPs, idiopathic interstitial pneumonias; KL-6, Krebs von den Lungen-6; LDH, lactate dehydrogenase; LTOT, long-term oxygen therapy; mMRC, modified Medical Research Council; NLR, neutrophil-to-lymphocyte ratio; PNI, prognostic nutritional index; PPFE, pleuroparenchymal fibroelastosis; SpO₂, saturation of arterial oxygen; UIP, usual interstitial pneumonia.

^a^ Those who could not measure their resting SpO_2_ with room air were included in resting SpO_2_ <95%

**Table S3.** Patient characteristics by FVC annual relative change after the initiation of nintedanib

|  | **FVC change ≥-1.37%** | **FVC change <-1.37%** | **p*-*value** |
| --- | --- | --- | --- |
|  | **n=107** | **n=107** |  |
| **Age** | 73.0 [38‒90] | 73.0 [20‒90] | 0.96 |
| **Male** | 82 (76.6) | 78 (72.9) | 0.64 |
| **BMI, kg/m^2^** | 23.9 [8.2‒31.4] | 22.3 [11.1‒36.8] | **0.005** |
| **Smoking status** |  |  |  |
| **Former/Current** | 73 (68.2) | 78 (72.9) | 0.13 |
| **Disease** |  |  |  |
| **IPF** | 42 (39.3) | 39 (36.4) | 0.80 |
| **IIPs** | 29 (27.1) | 31 (29.0) |  |
| **CTD-ILD** | 27 (25.2) | 23 (21.5) |  |
| **PPFE** | 4 (3.7) | 9 (8.4) |  |
| **HP** | 4 (3.7) | 4 (3.7) |  |
| **CT pattern** |  |  |  |
| **UIP** | 60 (56.1) | 60 (56.1) | 0.36 |
| **fNSIP** | 35 (32.7) | 35 (32.7) |  |
| **PPFE** | 3 (2.8) | 8 (7.5) |  |
| **cNSIP** | 2 (1.9) | 0 (0) |  |
| **OP** | 1 (0.9) | 0 (0) |  |
| **Unclassifiable** | 6 (5.6) | 4 (3.7) |  |
| **Past acute exacerbation** | 13 (12.1) | 12 (12.2) | 1.00 |
| **Medication** |  |  |  |
| **Glucocorticoid** | 37 (34.6) | 24 (22.4) | 0.07 |
| **Immunosuppressant** | 23 (21.5) | 10 (9.3) | **0.02** |
| **Severity Ⅲ, Ⅳ** | 39 (36.4) | 40 (37.4) | 0.68 |
| **mMRC 2‒4** | 43 (40.2) | 43 (40.2) | 1.00 |
| **Resting SpO_2_ <95% ^a^** | 24 (22.4) | 24 (22.4) | 1.00 |
| **LTOT** | 10 (9.3) | 13 (12.1) | 0.66 |
| **Blood test** |  |  |  |
| **ALB, g/dL** | 3.9 [2.7‒4.7] | 4.0 [3.0‒4.6] | 0.96 |
| **LDH, U/L** | 222.0 [142.0‒349.0] | 218.0 [156.0‒429.0] | 0.64 |
| **CRP, mg/dL** | 0.20 [0.00‒11.94] | 0.20 [0.03‒12.34] | 0.92 |
| **KL-6, U/mL** | 902.0 [169.0‒9450.0] | 988.5 [160.0‒5570.0] | 0.98 |
| **NLR** | 2.46 [0.77‒30.30] | 2.35 [0.78‒23.50] | 0.70 |
| **PNI** | 49.63 [29.65‒64.83] | 49.38 [36.24‒60.29] | 0.61 |
| **Pulmonary function test** |  |  |  |
| **%FVC** | 72.7 [23.6‒131.2] | 74.6 [33.7‒126.6] | 1.00 |
| **%DLco** | 54.2 [16.3‒119.8] | 59.0 [14.4‒105.5] | 0.51 |
| **Days from diagnosis to initiation of nintedanib** | 343.0 [-37‒5307] | 593.0 [-1164‒4005] | 0.30 |

Data are expressed as medians [ranges], or N (%).

%DLco, % diffusing capacity of the lungs for carbon monoxide; %FVC, % forced vital capacity; ALB, albumin; BMI, body mass index; cNSIP, cellular non-specific interstitial pneumonia; CRP, C-reactive protein; CTD-ILD, connective tissue disease-associated interstitial lung disease; fNSIP, fibrotic non-specific interstitial pneumonia; HP, hypersensitivity pneumonitis; IIPs, idiopathic interstitial pneumonias; IPF, idiopathic pulmonary fibrosis; KL-6, Krebs von den Lungen-6; LDH, lactate dehydrogenase; LTOT, long-term oxygen therapy; mMRC, modified Medical Research Council; NLR, neutrophil-to-lymphocyte ratio; OP, organizing pneumonia; PF-ILD, progressive fibrosing interstitial lung diseases; PNI, prognostic nutritional index; PPFE, pleuroparenchymal fibroelastosis; SpO₂, saturation of arterial oxygen; UIP, usual interstitial pneumonia.

^a^ Those who could not measure their resting SpO_2_ with room air due to oxygen administration were included in resting SpO_2_ <95%

**Table S4.** Patient characteristics by acute exacerbation within 3 years after the initiation of nintedanib

|  | **Without acute exacerbation** | **With acute exacerbation** | **p*-*value** |
| --- | --- | --- | --- |
|  | **n=114** | **n=62** |  |
| **Age** | 75.0 [20‒92] | 75.5 [59‒91] | 0.30 |
| **Male** | 89 (78.1) | 51 (82.3) | 0.56 |
| **BMI, kg/m^2^** | 20.8 [8.2‒32.6] | 22.5 [14.2‒31.5] | **0.02** |
| **Smoking status** |  |  |  |
| **Former/Current** | 89 (78.1) | 46 (74.2) | 0.68 |
| **Disease** |  |  |  |
| **IPF** | 50 (43.9) | 32 (51.6) | 0.66 |
| **IIPs** | 28 (24.6) | 15 (24.2) |  |
| **CTD-ILD** | 22 (19.3) | 10 (16.1) |  |
| **PPFE** | 9 (7.9) | 2 (3.2) |  |
| **HP** | 3 (2.6) | 3 (4.8) |  |
| **CT pattern** |  |  |  |
| **UIP** | 76 (66.7) | 40 (64.5) | 0.51 |
| **fNSIP** | 24 (21.1) | 18 (29.0) |  |
| **PPFE** | 8 (7.0) | 2 (3.2) |  |
| **Unclassifiable** | 6 (5.3) | 2 (3.2) |  |
| **Past acute exacerbation** | 14 (12.3) | 17 (27.4) | **0.02** |
| **Medication** |  |  |  |
| **Glucocorticoid** | 36 (31.6) | 28 (45.2) | 0.10 |
| **Immunosuppressant** | 18 (15.8) | 10 (16.1) | 1.00 |
| **Severity Ⅲ, Ⅳ** | 59 (51.8) | 26 (41.9) | 0.38 |
| **mMRC 2‒4** | 58 (50.9) | 30 (48.4) | 1.00 |
| **Resting SpO_2_ <95% ^a^** | 54 (47.4) | 22 (35.5) | 0.056 |
| **LTOT** | 28 (24.6) | 15 (24.6) | 1.00 |
| **Blood test** |  |  |  |
| **ALB, g/dL** | 3.8 [2.4‒5.0] | 3.7 [2.9‒4.6] | 0.76 |
| **LDH, U/L** | 235.0 [139.0‒818.0] | 232.0 [142.0‒514.0] | 0.83 |
| **CRP, mg/dL** | 0.39 [0.01‒25.73] | 0.30 [0.01‒4.12] | 0.20 |
| **KL-6, U/mL** | 910.0 [261.0‒5570.0] | 1104.0 [310.0‒3278.0] | 0.15 |
| **NLR** | 3.13 [0.33‒25.43] | 2.77 [0.77‒37.60] | 0.71 |
| **PNI** | 46.10 [14.40‒80.10] | 58.00 [23.30‒112.60] | 0.32 |
| **Pulmonary function test** |  |  |  |
| **%FVC** | 59.8 [23.6‒116.9] | 67.2 [40.9‒100.2] | 0.20 |
| **%DLco** | 46.1 [14.4‒80.1] | 48.3 [33.4‒59.1] | 0.22 |
| **Days from diagnosis to initiation of nintedanib** | 519.0 [-302‒5660] | 800.0 [0‒5096] | 0.10 |

Data are expressed as medians [ranges], or N (%).

%DLco, % diffusing capacity of the lungs for carbon monoxide; %FVC, % forced vital capacity; ALB, albumin; BMI, body mass index; CRP, C-reactive protein; CTD-ILD, connective tissue disease-associated interstitial lung disease; fNSIP, fibrotic non-specific interstitial pneumonia; HP, hypersensitivity pneumonitis; IIPs, idiopathic interstitial pneumonias; IPF, idiopathic pulmonary fibrosis; KL-6, Krebs von den Lungen-6; LDH, lactate dehydrogenase; LTOT, long-term oxygen therapy; mMRC, modified Medical Research Council; NLR, neutrophil-to-lymphocyte ratio; PF-ILD, progressive fibrosing interstitial lung diseases; PNI, prognostic nutritional index; PPFE, pleuroparenchymal fibroelastosis; SpO₂, saturation of arterial oxygen; UIP, usual interstitial pneumonia.

^a^ Those who could not measure their resting SpO_2_ with room air due to oxygen administration were included in resting SpO_2_ <95%

**Table S5.** Multivariate analysis of patient characteristics predicting acute exacerbation within 3 years after the initiation of nintedanib

|  | **Odds ratio** | **95% confidence interval** | ***p-*value** |
| --- | --- | --- | --- |
| **Age** | 1.020 | 0.99‒1.07 | 0.22 |
| **Male** | 1.230 | 0.53‒2.85 | 0.63 |
| **BMI** | 1.100 | 1.02‒1.19 | **0.02** |
| **Past acute exacerbation** | 2.840 | 1.24‒6.55 | **0.01** |

BMI: body mass index.

**Table S6.** Nintedanib usage condition

|  | **Survival <3 year** | **Survival ≥3 year** | **p*-*value** |
| --- | --- | --- | --- |
|  | **n=155** | **n=106** |  |
| **Starting dose** |  |  |  |
| **200mg/day** | 60 (38.7) | 36 (34.0) | 0.52 |
| **300mg/day** | 86 (55.5) | 66 (62.3) |  |
| **Maximum dose** |  |  |  |
| **200mg/day** | 58 (37.4) | 24 (22.6) | **0.03** |
| **300mg/day** | 92 (59.4) | 79 (74.5) |  |
| **Duration, days** | 257.0 [1‒1108] | 1326.0 [13‒1822] | **<0.001** |
| **Discontinuation** | 69 (44.5) | 31 (29.2) | **0.01** |
| **Reason of discontinuation** |  |  |  |
| **Adverse event** | 38 (55.1) | 18 (56.2) | 0.69 |
| **Worsening of ILD** | 10 (14.5) | 3 (9.4) |  |
| **Poor physical condition** | 8 (11.6) | 2 (6.2) |  |
| **Surgery, Pneumothorax, Injury** | 4 (5.8) | 4 (12.5) |  |
| **others** | 9 (13.0) | 5 (15.6) |  |

Data are expressed as medians [ranges], or N (%).

ILD, interstitial lung disease.

**Supplementary Figure S1.**


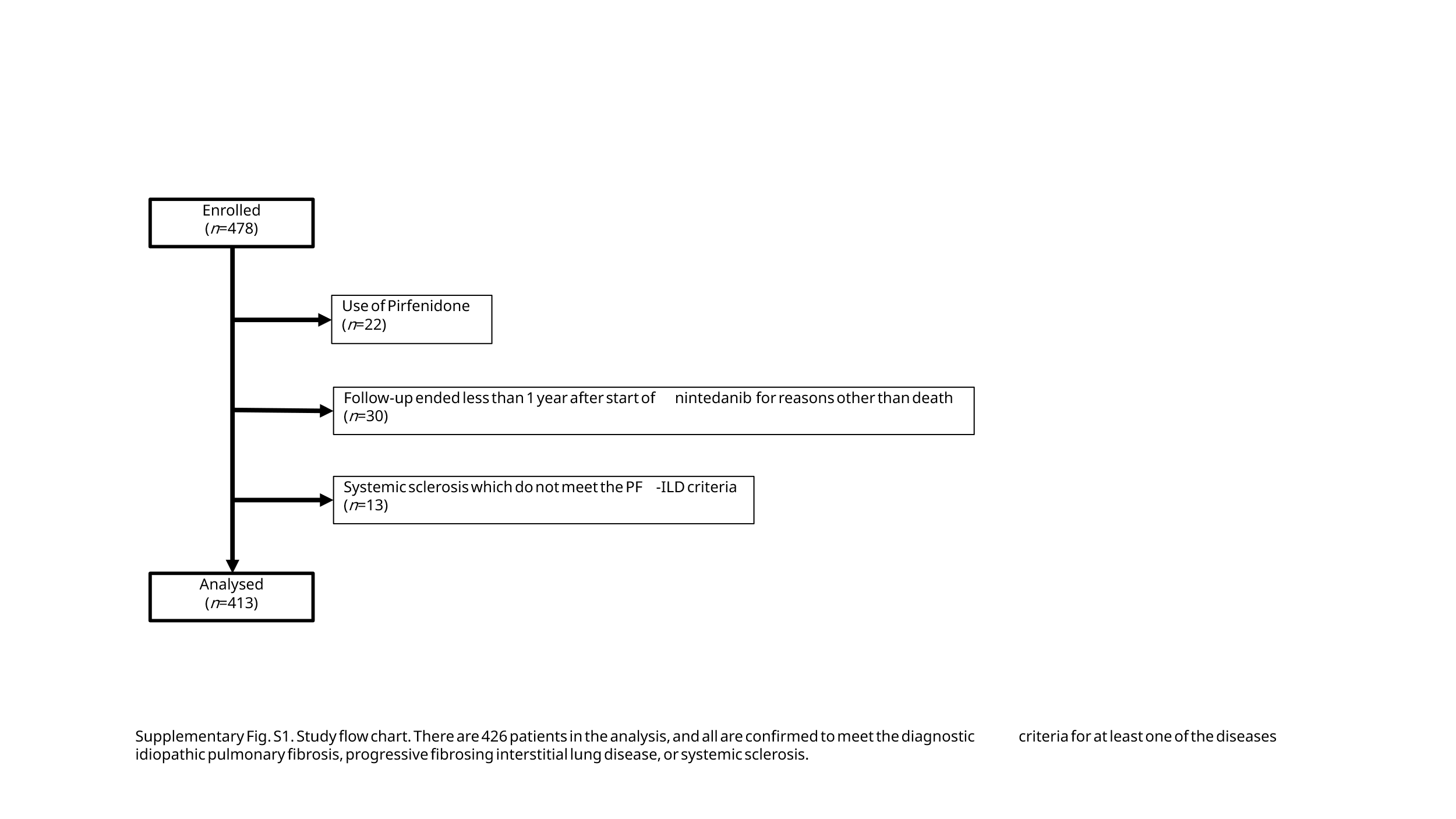


**Supplementary Figure S2.**

**
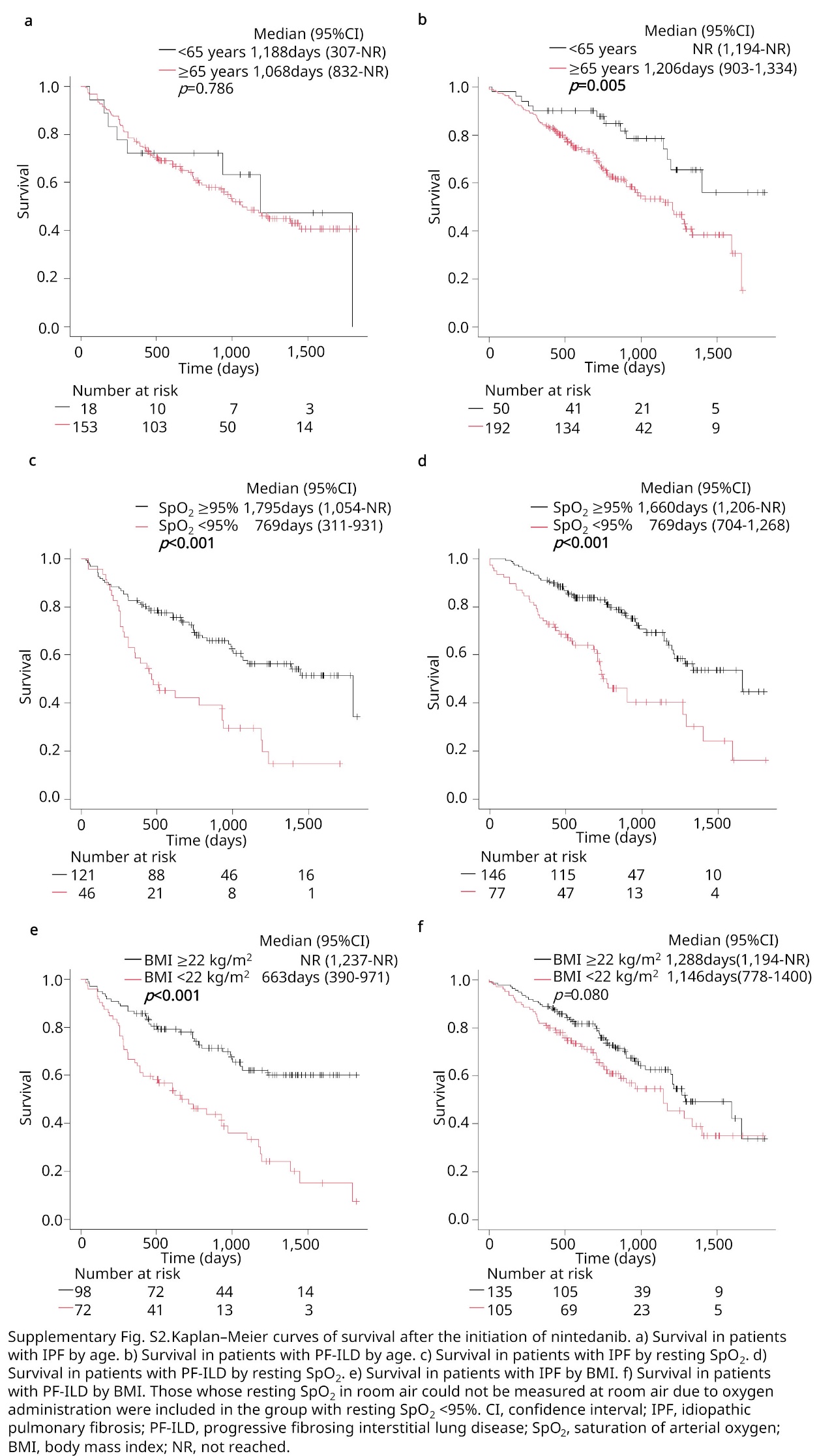
**

**Supplementary Figure S3.**


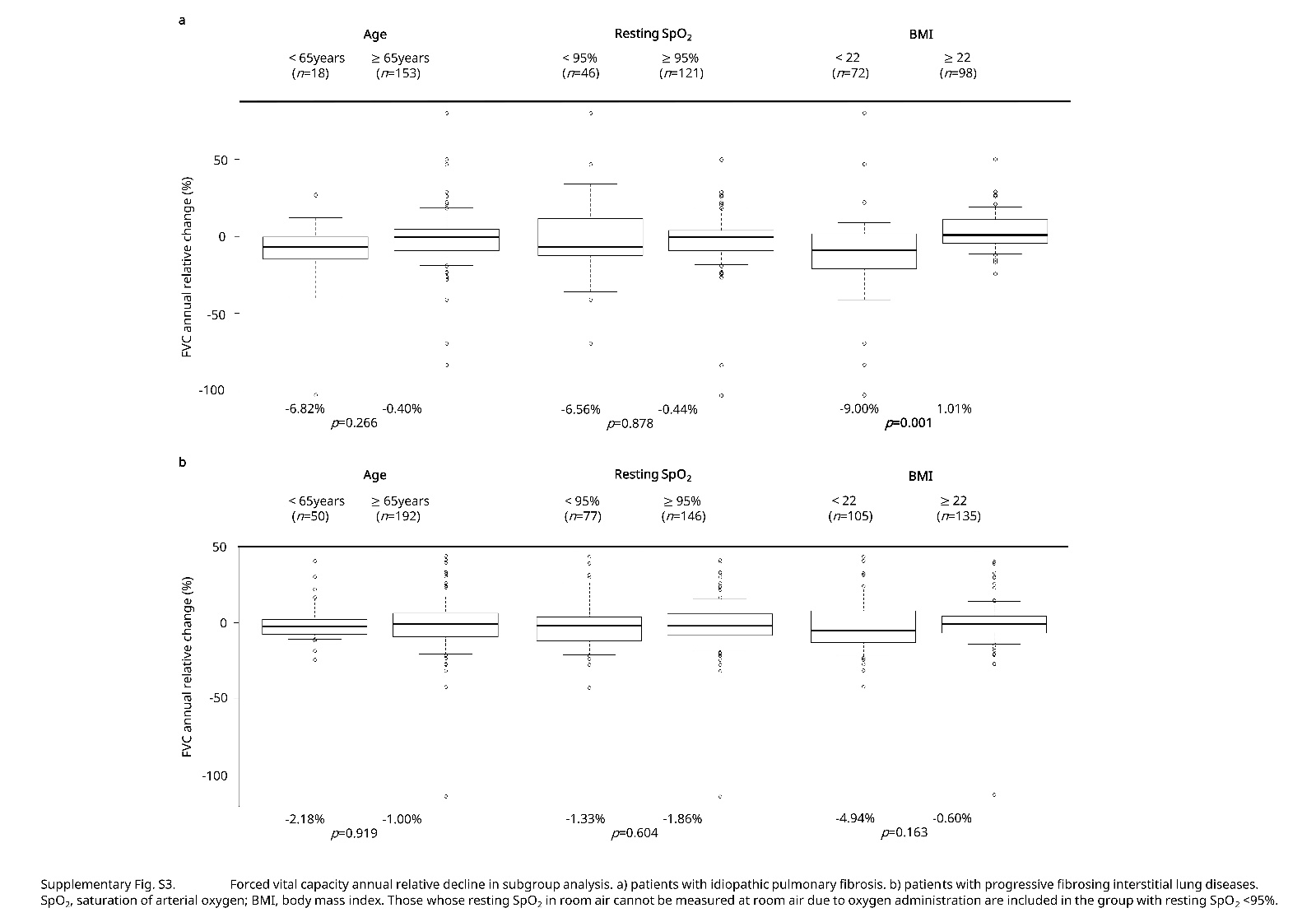


**Supplementary Figure S4.**


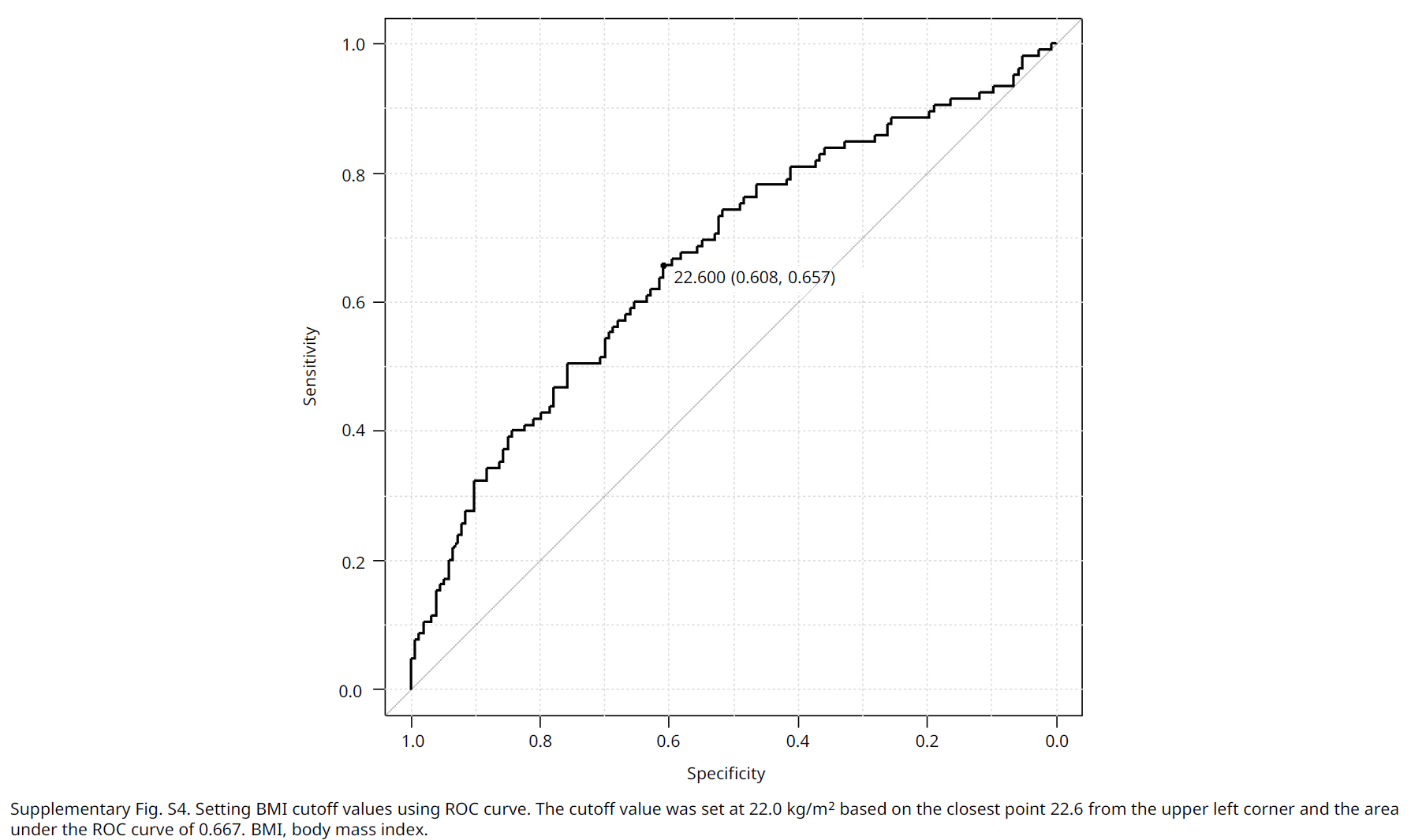

Supplement: Supplementary file 1 — Supplementary Material 1 [file 41598_2025_34071_MOESM1_ESM.docx]
